# Role of Rho kinase and Na^+^/H^+^ exchange in hypoxia‐induced pulmonary arterial smooth muscle cell proliferation and migration

**DOI:** 10.14814/phy2.12702

**Published:** 2016-03-23

**Authors:** Jasmine Walker, Clark Undem, Xin Yun, Julie Lade, Haiyang Jiang, Larissa A. Shimoda

**Affiliations:** ^1^ Division of Pulmonary and Critical Care MedicineDepartment of MedicineJohns Hopkins School of MedicineBaltimoreMaryland

**Keywords:** Pulmonary hypertension, remodeling, vascular

## Abstract

Abnormal proliferation and migration of pulmonary arterial smooth muscle cells (PASMCs) are hallmark characteristics of vascular remodeling in pulmonary hypertension induced by chronic hypoxia. In this study, we investigated the role of the Na^+^/H^+^ exchanger (NHE) and alterations in intracellular pH (pH_i_) homeostasis in meditating increased proliferation and migration in PASMCs isolated from resistance‐sized pulmonary arteries from chronically hypoxic rats or from normoxic rats that were exposed to hypoxia ex vivo (1% or 4% O_2_, 24–96 h). We found that PASMCs exposed to either in vivo or ex vivo hypoxia exhibited greater proliferative and migratory capacity, elevated pH_i_, and enhanced NHE activity. The NHE inhibitor, ethyl isopropyl amiloride (EIPA), normalized pH_i_ in hypoxic PASMCs and reduced migration by 73% and 45% in cells exposed to in vivo and in vitro hypoxia, respectively. Similarly, EIPA reduced proliferation by 97% and 78% in cells exposed to in vivo and in vitro hypoxia, respectively. We previously demonstrated that NHE isoform 1 (NHE1) is the predominant isoform expressed in PASMCs. The development of hypoxia‐induced pulmonary hypertension and alterations in PASMC pH_i_ homeostasis were prevented in mice deficient for NHE1. We found that short‐term (24 h) ex vivo hypoxic exposure did not alter the expression of NHE1, so we tested the role of Rho kinase (ROCK) as a possible means of increasing NHE activity. In the presence of the ROCK inhibitor, Y‐27632, we found that pH_i_ and NHE activity were normalized and migration and proliferation were reduced in PASMCs exposed to either in vivo (by 68% for migration and 22% for proliferation) or ex vivo (by 43% for migration and 17% for proliferation) hypoxia. From these results, we conclude that during hypoxia, activation of ROCK enhances NHE activity and promotes PASMC migration and proliferation.

## Introduction

Persistent alveolar hypoxia is associated with many chronic lung diseases, and results in the development of pulmonary hypertension, significantly impacting patient morbidity and mortality. Previous studies have determined that the elevation in pulmonary arterial pressure observed in response to prolonged hypoxia arises from sustained contraction and structural remodeling of the small arteries in the pulmonary circulation. Despite efforts to better understand the changes that occur in the pulmonary vasculature during chronic hypoxia (CH), the exact cellular mechanisms underlying these changes are not completely understood.

Several studies have proposed an important role for changes in pulmonary arterial smooth muscle cell (PASMC) intracellular pH (pH_i_) in the pathogenesis of hypoxic pulmonary hypertension (Huetsch and Shimoda 2015). Regulation of pH_i_ is vital for maintaining cell viability, as changes in pH_i_ are associated with a number of important cell functions, including volume regulation, signal transduction pathways involved in cell proliferation, and mediator release. Most mammalian systems possess three mechanisms by which pH_i_ homeostasis is maintained. These include the Na^+^‐dependent Cl^−^/HCO_3_ exchanger, the Na^+^‐independent Cl^−^/HCO_3_ exchanger, and the Na^+^/H^+^ exchanger (NHE). All of these exchangers have been shown to exist in vascular smooth muscle (Aalkjaer and Cragoe 1988; Aalkjaer and Mulvany 1988; Rao et al. 1990; Lucchesi et al. 1994; Lucchesi and Berk 1995; Aalkjaer and Peng 1997), with NHE playing a substantial role in controlling PASMC pH_i_ (Quinn et al. 1991; Madden et al. 2001; Rios et al. 2005). NHEs reside in cell membranes and use the transmembrane Na^+^ gradient to extrude protons. The importance of NHE in the pulmonary circulation is evidenced by studies demonstrating that activation of NHE and alkalinization are required for PASMC proliferation in response to growth factors (Quinn et al. 1996).

Both acute hypoxia (Madden et al. 2001) and CH (Rios et al. 2005; Shimoda et al. 2006) increase pulmonary vascular smooth muscle pH_i_. We previously demonstrated that the alkaline shift in pH_i_ observed in PASMCs isolated from chronically hypoxic rats and mice is due to upregulation of NHE isoform 1 (NHE1) and consequently, enhanced NHE activity (Rios et al. 2005; Shimoda et al. 2006). Consistent with a role for NHE in PASMC growth in this model of hypoxia‐induced pulmonary hypertension, when NHE was inhibited with pharmacologic agents (Quinn et al. 1998) or when NHE1 was genetically deleted in mice (Yu et al. 2008), both vascular remodeling and development of pulmonary hypertension in response to CH were attenuated. More recent experiments using PASMCs cultured from human proximal pulmonary arteries demonstrated that silencing NHE1 was sufficient to prevent the proliferation and migration induced by exposure to severe hypoxia in vitro (Yu and Hales 2011). These data strongly suggest that activity and/or expression of NHE regulates hypoxia‐induced proliferation and/or migration of PASMCs. A caveat to these experiments is that it is unknown whether inhibiting NHE after pulmonary hypertension is established can slow or reverse the proliferative and migratory phenotype of PASMCs or whether the mechanisms governing hypoxia‐induced growth are similar under conditions of severe (i.e., 1% O_2_) and moderate (i.e., 4% O_2_) hypoxia. Also, it has been established that PASMCs derived from proximal and distal sites in the lung can exhibit marked differences in growth responses (Wharton et al. 2000; Yang et al. 2005). Thus, it is important to also determine whether inhibiting NHE activity in PASMCs from the distal vasculature, the most prominent site of hypoxia‐induced vascular remodeling, is an effective means to reduce growth responses.

Our previous studies showed that activation of Rho kinase (ROCK) by the vasoconstrictor, endothelin‐1, can enhance NHE activity in PASMCs, leading to alkalinization of pH_i_ (Undem et al. 2012). Increased PASMC ROCK expression and activity in response to CH has been demonstrated in a variety of studies (McMurtry et al. 2003; Girgis et al. 2007; Jernigan et al. 2008; de Frutos et al. 2011), and thus, may facilitate enhanced NHE activity. Therefore, in this study, we also tested the hypothesis that hypoxia‐induced proliferation and migration of PASMCs isolated from the distal vasculature require ROCK‐mediated increases in NHE activity.

Using an in vitro model consisting of PASMCs isolated from CH rats, as well as PASMCs isolated from normal rats and exposed to hypoxia ex vivo, we assessed the effects of prolonged hypoxia on PASMC pH_i_, NHE activity, migration, and proliferation, and the role of ROCK in mediating these changes. We also used an in vivo model to evaluate the effect of NHE1 deficiency on the development of hypoxic pulmonary hypertension, vascular remodeling, and pH homeostasis.

## Material and Methods

### In vivo exposure to hypoxia

All protocols were approved by the Johns Hopkins Animal Care and Use Committee. Adult male Wistar rats (250–300 g) were obtained from Harlan farms. Adult (6‐ to 8‐week‐old) male mice with deficiency for NHE1 (*Nhe1* ^*−/−*^; strain # B6.SJL‐Slc91^swe^/J) were purchased from Jackson labs. Since this is a spontaneous mutation, age‐matched male C57B/6 mice were used as wild‐type (*Nhe1* ^*+/+*^) controls. Mice or rats were placed in a hypoxic chamber for 3 weeks as described previously (Rios et al. 2005). The chamber was continuously gassed with a mixture of room air and 100% N_2_ to maintain 10 ± 0.5% O_2_ and low CO_2_ concentrations (0.5%). A servo control mechanism was used to inject N_2_ as required and continuously monitor O_2_ levels inside the chamber (PRO‐OX; RCI Hudson, Anaheim, CA). Animals had free access to food and water and were removed from the chamber for less than 5 min twice a week to replenish food and water supplies and clean the cages. Because some assays required that measurements in cells from normoxic and hypoxic animals be made simultaneously, the normoxic control animals were housed next to the chamber in room air and exposed to the same light–dark cycle and ambient temperatures. Humidity and temperature within the chamber were monitored daily and were similar to that experienced by the room air animals. Although our chamber was continuously flushed, the flow rate was quite low, and we believe it is unlikely that there were any significant environmental differences between the two exposure groups; however, we acknowledge that the exposure conditions were not completely identical.

### Assessment of vascular remodeling

Lungs were fixed via tracheal instillation of 10% formalin. The right lung was tied off for isolation of vessels, and 0.5 mL of formalin was instilled into the left lung of each animal. The thorax was opened, and the heart and lungs were removed en bloc. The lungs were immersed and stored in 10% buffered formalin for sectioning. Fixed lungs were transferred to 70% ethanol, embedded in paraffin, and sectioned into 5‐*μ*m slices parallel to the hilum. Sections from each lung were stained with a monoclonal antibody directed against smooth muscle cell specific *α*‐actin (SMA) and counterstained with hematoxylin. For each lung, 20 nonoverlapping images were collected via an Olympus camera mounted on the microscope and connected to Q‐capture software. Each section was analyzed for vessels with an external diameter ≤100 *μ*m using Image J software (NIH, Bethesda, MD) calibrated with a 0.01‐mm microscope stage micrometer calibration slide. After vessels were identified as ≤100 *μ*m outer diameter, each vessel was classified as actin positive (≥50% circumferential staining for SMA) or negative. All histology was performed by an investigator blinded to genotype and treatment group.

### Isolation of pulmonary arterial smooth muscle cells

The method for obtaining primary cultures of murine and rat PASMCs has been described previously (Rios et al. 2005; Shimoda et al. 2006). Briefly, animals were anesthetized with sodium pentobarbital (130 mg/kg i.p.) and the heart and lungs were removed en bloc and placed in HEPES‐buffered salt solution (HBSS) containing (in mmol/L): 130 NaCl, 5 KCl, 1.2 MgCl_2_, 1.5 CaCl_2_, 10N‐[2‐hydroxyethyl]piperazine‐N′‐[2‐ethanesulfonic acid] (HEPES), and 10 glucose, with pH adjusted to 7.2 with 5 mol/L NaOH. The atria were removed and the right ventricle (RV) of the heart was separated from the left ventricle and the septum (LV+S) and the two portions blotted dry and weighed. Distal (≥5th generation) intrapulmonary arteries (200–500 *μ*m o.d.) were dissected free from connective tissue in ice‐cold HBSS. The arteries were opened and the lumen gently rubbed to remove the endothelium. Tissue was allowed to recover for 30 min in cold (4°C) HBSS followed by 20 min in reduced Ca^2+^ HBSS (20 *μ*mol/L CaCl_2_) at room temperature. The tissue was digested (20 min for rat; 10 min for mouse) at 37°C in an enzyme solution consisting of reduced Ca^2+^ HBSS with collagenase (type I; 1750 U/mL), papain (9.5 U/mL), bovine serum albumin (2 mg/mL), and dithiothreitol (1 mmol/L). After digestion, trituration with a wide‐bore transfer pipette was used to disperse single smooth muscle cells in Ca^2+^‐free HBSS. The cell suspension was placed on 25‐mm glass cover slips and cultured for 2–3 days. Rat PASMCs were cultured in Ham's F‐12 media supplemented with 0.5% FCS and 1% penicillin/streptomycin. Murine PASMCs were cultured in SmGm Complete Media (Lonza, Walkersville, MD) supplemented with 10% fetal calf serum for 2 days and placed in serum‐free media 12–24 h before experiments.

### Right ventricular systolic pressure and hypertrophy

Closed‐chest RV pressure was measured in anesthetized mice through an abdominal incision as described previously (Yu et al. 1999; Abud et al. 2012). Mice were lightly anesthetized with sodium pentobarbital (60 mg/kg), the diaphragm visualized through the abdomen, and RV pressure measured using a 23‐gauge needle filled with heparinized saline and connected to a pressure transducer (model P10EZ; Spectramed, Oxnard, CA). Correct localization of the puncture was verified by postmortem inspection. Pressure was recorded using Power Lab Software (ADI Instruments, Colorado Springs, CO). Only mice in which stable tracings were obtained and right ventricular puncture was verified were included in the analysis. Mean RV pressure was calculated as: 2/3 diastolic pressure + 1/3 systolic pressure and was averaged from data for at least five heartbeats. Following pressure measurements, the hearts were removed and the atria and extraneous vascular material were removed from the heart under a dissecting microscope. The RV wall was carefully separated from the left ventricle and the septum (LV+S), and both portions were quickly blotted dry and weighed.

### In vitro exposure to hypoxia

Pulmonary arterial smooth muscle cells were exposed to hypoxia ex vivo by placing cells in an airtight chamber (Billups‐Rothenberg, Del Mar, CA) gassed with 4% O_2_; 5% CO_2_ or 1% O_2_; 5% CO_2_. The chamber was flushed prior to beginning exposure and again at 48 h. The chamber and the nonhypoxic controls (20% O_2_; 5% CO_2_) were placed in an incubator at 37°C for 60 h. Initial experiments were performed using a hand‐held oxygen monitor (Model 5577; Hudson RCI) to assure that the chamber was able to sustain the desired level of hypoxia for a minimum of 48 h.

### pH_i_ measurements

Pulmonary arterial smooth muscle cells were placed in a laminar flow cell chamber perfused with modified Kreb's solution (KRBS) containing (in mmol/L):118.3 NaCl, 4.7 KCl, 1.2 MgSO_4_, 25 NaHCO_3_, 11 glucose, and 1.2 KH_2_PO_4_, and gassed with 16% O_2_; 5% CO_2_, or with HBSS with pH adjusted to 7.4 as described previously (Rios et al. 2005; Shimoda et al. 2006). Cells were incubated with the membrane permeant (acetoxymethyl ester) form of the pH‐sensitive fluorescent dye 2′,7′‐bis(carboxyethyl)‐5(6)‐carboxyflourescein (BCECF‐AM; Life Technologies) for 60 min at 37°C under an atmosphere of 20% O_2_; 5% CO_2_. Cells were then washed with KRBS or HBSS for 15 min at 37°C to remove extracellular dye and allow complete de‐esterification of cytosolic dye. Ratiometric measurement of BCECF fluorescence was performed on a workstation (Intracellular Imaging Inc, Cincinnati, OH) consisting of a Nikon TSE 100 Ellipse inverted microscope with epifluorescence attachments. Light from a xenon arc lamp was alternately filtered by 490 and 440 nm interference filters, and focused onto PASMCs via a 20× fluorescence objective (Super Fluor 20; Nikon, Melville, NY). A filter cube was used to collect light emitted from the cell at 530 nm. Filtered light was then returned through the objective and detected by an imaging camera. Between measurements, an electronic shutter (Sutter Instruments) was used to minimize photobleaching of the dye. All protocols were performed and data collected online with InCyte software (Intracellular Imaging Inc). The ratio of 490 to 440 nm emission was calculated and pH_i_ was estimated from in situ calibration after each experiment. For calibration, cells were perfused with a high K^+^ solution containing (in mmol/L): 105 KCl, 1 MgCl_2_, 1.5 CaCl_2_, 10 glucose, 20 HEPES‐Tris, and 0.01 nigericin to allow pH_i_ to equilibrate to external pH. A 2‐point calibration was created from fluorescence measured with pH_i_ adjusted to 6.5 and 7.5. The formula: pH_i_ = −log ([H^+^]_i_) was used to determine intracellular H^+^ ion concentration ([H^+^]_i_) from pH_i_.

### Cell migration

Equal numbers of cells (50,000) were seeded onto 24‐mm polystyrene transwell inserts with transparent, permeable (8 *μ*mol/L pore) filters. Cells were placed in 3 mL of Ham's F‐12 media supplemented with 0.5% FCS and 1% penicillin/streptomycin. Cells were allowed to adhere for 1–2 h before addition of drugs or beginning hypoxic exposures. After 24 h, filters were washed with PBS, and cells fixed in ice‐cold 95% EtOH for 10 min. Following fixation, cells were washed with PBS and stained with Coomassie blue for 5 min at room temperature. Filters were washed and placed in PBS. Each filter was examined under 80× magnification and images of five fields per filter were obtained at random. The top of the filters were then scraped with a cotton swab, rinsed with PBS to remove nonadherent cells, and images of five fields containing migrated cells were again obtained at random. Once images of all fields were obtained, the number of cells in each image was counted. Cell migration was expressed as the number of migrated cells as a percent of the total cells.

### Cell proliferation

Initial experiments were performed to compare proliferation measured via cell counts and via enzyme‐linked immunosorbent assay (ELISA) for BrdU incorporation. Cells were isolated and plated for 2–3 days to allow adherence of healthy cells. Cells were then washed, trypsinized, resuspended in media, and counted with a hemocytometer. For cell counts, 100,000 cells were plated in a 60‐mm diameter cell culture dish containing 3 mL Ham's F‐12 media supplemented with 0.5% FCS and 1% penicillin/streptomycin. Cells were incubated under control or hypoxic conditions for 72 h. At the end of exposure, cells were washed with PBS, trypsinized, cells counted using a hemocytomer, and total cell number per dish calculated. Each condition was performed in duplicate and values from the two dishes averaged for each experiment. For cell proliferation measured via ELISA, equal numbers of cells (5000/well) isolated from either normoxic or chronically hypoxic rats were placed in the wells of a 96‐well plate, with each condition repeated in triplicate. Ham's F‐12 media containing 0.5% FCS and 1% penicillin/streptomycin was added to each well for a total volume of 200 *μ*L. Cells were incubated for 1 h before treatments (10^−5^ mol/L EIPA or vehicle) were added. Cells were incubated at 37°C under control (20% O_2_; 5% CO_2_) or hypoxic (1 or 4% O_2_; 5% CO_2_) conditions for 72 h before addition of 20 *μ*L of labeling BrdU. Cells were allowed to incubate for additional 24 h in the presence of BrdU. Incorporation of BrdU into proliferating cells was measured using an ELISA (Biotrak System; GE Healthcare, Pittsburgh, PA) according to manufacturer's instructions. After 10 min of color development, reactions were stopped and well values were measured at 450 nm with a plate reader. Each sample and treatment was run in triplicate and the values were averaged.

### Protein isolation and immunoblot analysis

Cells were lysed in ice‐cold lysis buffer consisting of T‐PER buffer (Pierce, Grand Island, NY) containing protease inhibitor cocktail (Roche Diagnostics, Indianapolis, IN). Total protein samples were homogenized by sonication before being assayed for total protein content using the BCA protein assay (Pierce). Proteins (10 mg/lane) were separated by SDS‐PAGE and transferred to polyvinylidene difluoride membranes, which were probed with primary antibody directed against NHE1 (EMD Millipore, Billerica, MA) and goat anti‐mouse secondary antibody and visualized by ECL. For all experiments, membranes were probed for NHE1, stripped, and reprobed for the housekeeping protein (*β*‐tubulin; loading control).

### RNA isolation, reverse transcription, and real‐time PCR

Total RNA was prepared from samples using the RNeasy extraction kit with on‐column DNase treatment (Qiagen, Valencia, CA). Total RNA (500 ng) was reverse transcribed using the iScript cDNA synthesis kit (BioRad, Hercules, CA). Real‐time PCR was performed on an iCyclerIQ thermocylcer (BioRad) using 50 ng of the first‐strand cDNA mixture and 250 nmol/L forward and reverse primers in a 25‐*μ*L reaction volume containing QuantiTect SYBR Green PCR Master Mix (Qiagen). Specific primers were designed from sequences of the coding regions corresponding to the genes of interest and validated as described previously (Pisarcik et al. 2013). Transcripts of both NHE1 and cyclophilin B (CpB; housekeeping) were amplified for 45 cycles by annealing at 58°C for 20 sec, extending at 72°C for 20 sec, and denaturing at 94°C for 15 sec. At the end of the run, melt curves were performed to confirm amplification of a single product. Comparisons between treatments were always run on the same 96‐well plate. Quantification of each mRNA was calculated using the Pfaffl method (Pfaffl 2001). PCR detection threshold cycle (*C*_T_) values for each plate were calculated using iCylcer software and the efficiency of each primer pair determined from a 5‐point standard curve for each mRNA of interest. Data were expressed as a ratio of NHE1 mRNA to CpB within a sample.

### Drugs and chemicals

All chemicals were obtained from Sigma (ST. Louis, MO).

### Statistical analysis

Each experiment was conducted in cells from different animals. Thus, the number of experiments also refers to the number of animals in each protocol. For experiments where pH_i_ was measured, each experiment consisted of 10–30 cells, with the individual values for each cell averaged to get a mean value for the experiment. For statistical comparisons, pH values were converted to [H^+^] before running statistics. Comparisons between groups were made using one‐way or two‐way analysis of variance as appropriate, with Holm–Sidak posttest for differences between groups. A *P *<* *0.05 was accepted as statistically significant.

## Results

### Effect of CH on pH homeostasis in rat PASMCs

**Figure 1:**
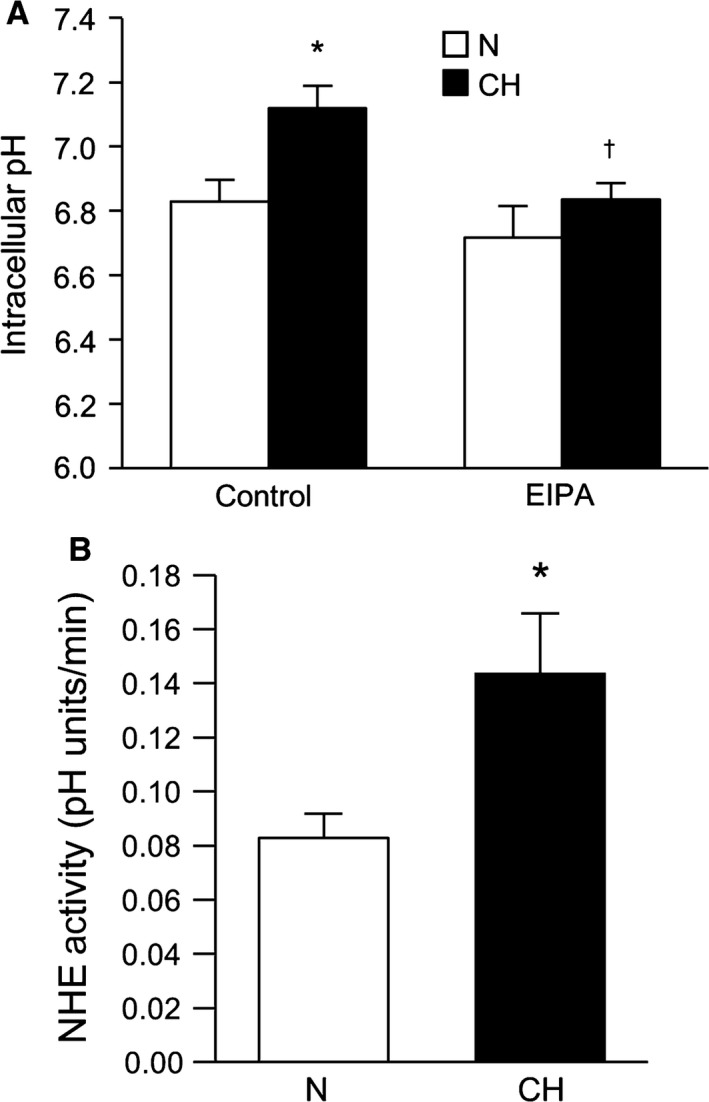
Exposure to chronic hypoxia increases baseline intracellular pH (pH_i_) and Na^+^/H^+^ exchange (NHE) activity in pulmonary arterial smooth muscle cells (PASMCs). (A) Bar graphs illustrating basal pH_i_ measured in the absence and presence of the NHE inhibitor, ethyl isopropyl amiloride (EIPA) in PASMCs isolated from normoxic (*n* = 14 for control and *n* = 4 for EIPA) and chronically hypoxic (CH;* n* = 7 for control and *n* = 5 for EIPA) rats during perfusion with HEPES‐buffered extracellular solution. (B) Bar graphs illustrating values for Na^+^‐dependent recovery rates from acidosis after 2 min (NHE activity) were compared in PASMCs isolated from normoxic (*n* = 14) and CH (*n* = 9) rats. All bar graphs show mean ± SEM data. *Indicates significant difference from control normoxic value (*P *< 0.05); †indicates significant difference from control hypoxic value.

We previously demonstrated that pH_i_ was more alkaline in PASMCs derived from mice exposed to CH (Rios et al. 2005). In PASMCs isolated from rats exposed to CH perfused with a bicarbonate‐free extracellular solution average baseline pH_i_ was also significantly higher compared to that measured in PASMCs from normoxic rats (Fig. 1A), confirming our previous findings in mice. As expected, application of the NHE inhibitor, EIPA (10^−5^ mol/L), caused a small reduction in basal pH_i_ in PASMCs from normoxic rats (*P *< 0.05) and decreased pH_i_ in PASMCs from chronically hypoxic rats to a greater extent (*P *< 0.05). Consistent with the observed CH‐induced increase in basal pH_i_, we found that NHE activity measured using the ammonium pulse technique, was significantly increased in PASMCs from CH rats (Fig. 1B).

### Effect of CH on PASMC migration

**Figure 2:**
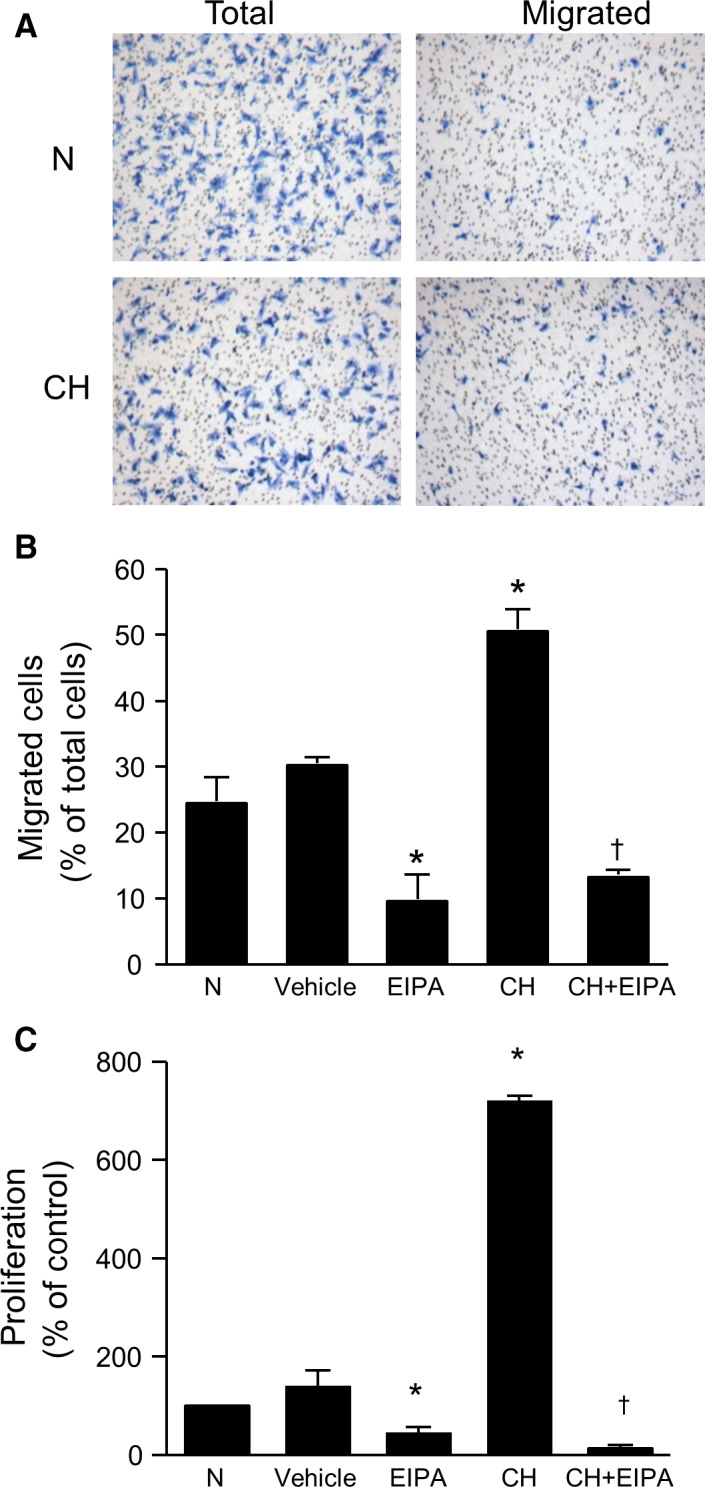
EIPA attenuates CH‐induced migration and proliferation of PASMCs. (A) Representative images showing PASMC migration assessed by transwell assay. Panels show total number of cells on the membrane before and after scraping the top chamber (unmigrated cells) for cells from normoxic (N) and chronically hypoxic (CH) rats. (B) Bar graphs illustrating percent of migrated cells isolated from N and CH rats with (*n* = 6 for N and *n* = 4 for CH) and without EIPA (10^−5^ mol/L; *n* = 15 for N and *n* = 5 for CH), a NHE inhibitor. Vehicle treatment had no effect on migration (*n* = 4). (C) Bar graphs illustrating in vitro proliferation of PASMCs with and without EIPA (10^−5^ mol/L) isolated from N (*n* = 23 for untreated and *n* = 12 for EIPA) and CH (*n* = 8 each for untreated and EIPA) rats. Vehicle control (*n* = 4) had no significant effect on proliferation. Proliferation values are presented as percent of N. All bar graphs show mean ± SEM data. *Indicates significant difference from N value (*P *< 0.05); †indicates significant difference from CH value.

We next determined the effect of CH on PASMC migratory capacity. Using the transwell migration assay, we found that PASMCs isolated from normoxic animals exhibited a basal migration rate of 23.9 ± 2.1% (*n* = 13) after 24 h (Fig. 2A and B). Vehicle treatment (1:10,000 DMSO) had neither significant effect on basal migration rates nor cell adherence (431.5 ± 36.6 cells/5 fields for control vs. 377.5 ± 79.1 cells/5 fields for vehicle). EIPA (10^−5^ mol/L) markedly reduced basal migration, but did not alter adherence (390 ± 35.3 cells/5 fields). Adherence of PASMCs from chronically hypoxic rats to the membrane was not statistically different from normoxic PASMCs (318 ± 50.5 cells/5 fields), but the rate of migration was significantly higher (Fig. 2A and B) and the CH‐induced increase in migratory ability was abolished in the presence of EIPA.

### Effect of CH on PASMC proliferation

In initial experiments, proliferation results obtained with ELISA were validated by comparing the results against those obtained using cell counts. Cells were placed in control conditions or challenged with 10% FBS. In the presence of FBS, cell proliferation measured by cell counts was 813.8 ± 300% (*n* = 5). This value compared favorably with values obtained using the ELISA (877 ± 281% of control; *n* = 13). Similar comparable results between methods were obtained for PASMCs exposed to CH and to 1% O_2_ (data not shown). The role of NHE in CH‐induced PASMC proliferation was then determined by performing ELISA on cells isolated from both normoxic and chronically hypoxic rats in the presence and absence of EIPA (10^−5^ mol/L). PASMCs isolated from chronically hypoxic rats exhibited an increase in proliferation compared to PASMCs from normoxic rats (Fig. 2C). EIPA markedly reduced proliferation in PASMCs from both normoxic and chronically hypoxic rats. In contrast, vehicle treatment (1:10,000 DMSO) had no significant effect on proliferation.

### Effect of in vitro hypoxia on pH homeostasis

**Figure 3:**
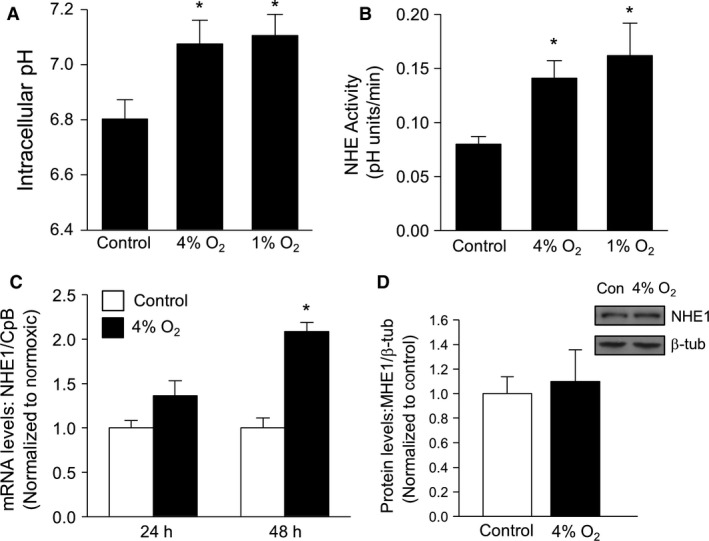
Exposure to in vitro hypoxia increases basal pH_i_ and NHE activity, but not NHE1 expression, in PASMCs. (A) Bar graphs illustrating average basal pH_i_ measured in PASMCs during perfusion with HEPES‐buffered extracellular solution after exposure for 24 h to control conditions (*n* = 12), 4% O_2_ (*n* = 6), or 1% O_2_ (*n* = 7). (B) Bar graphs illustrating mean values for Na^+^‐dependent recovery rates from acidosis at 2 min compared in PASMCs exposed to control conditions (*n* = 12), 4% O_2_ (*n* = 6), or 1% O_2_ (*n* = 5). (C) Bar graph showing NHE1 mRNA expression (normalized to cyclophilin B) in PASMCs exposed to control conditions (*n* = 4 for 24 and *n* = 5 for 48 h) or 4% O_2_ (*n* = 4 for 24 and *n* = 4 for 48 h). (D) Representative blot and bar graph showing NHE1 protein expression in PASMCs exposed to control conditions (*n* = 3) or 4% O_2_ (*n* = 3) for 24 h. All bar graphs show mean ± SEM data. *Indicates significant difference from N value (*P *< 0.05).

Pulmonary arterial smooth muscle cells isolated from normoxic rats were placed in a modular incubator in an atmosphere of 4% or 1% O_2_ for 24 h before measurements of basal pH_i_ and NHE activity were obtained. In nonhypoxic cells, average pH_i_ (Fig. 3A) was similar to that observed in our previous studies (Rios et al. 2005). Exposure to either 1% O_2_ or 4% O_2_ significantly increased basal pH_i_. Application of EIPA caused a significant reduction in basal pH_i_ in cells exposed to either 4% O_2_ (−0.1 ± 0.01 pH units; *n* = 5 experiments) or 1% O_2_ (−0.09 ± 0.03 pH units; *n* = 4 experiments). NHE activity in nonhypoxic cells (Fig. 3B) was similar to values measured previously in our laboratory (Shimoda et al. 2006; Undem et al. 2012), and was significantly increased by ex vivo exposure to hypoxia. These results confirm that short‐term exposure to in vitro hypoxia directly alters pH homeostasis in rat PASMCs.

We previously demonstrated that exposure to 4% O_2_ for 48 or 60 h induced an increase in NHE1 expression, both at the level of mRNA and protein (Shimoda et al. 2006; Pisarcik et al. 2013). To determine whether the changes in pH homeostasis that we observed after 24 h of hypoxic exposure were associated with changes in NHE1 expression, real‐time RT‐PCR and immunoblot analysis were performed. Despite increasing PASMC pH_i_ and NHE activity, exposure to 4% O_2_ had no significant effect on NHE1 mRNA levels at 24 h (Fig. 3C), whereas a significant increase in NHE1 mRNA levels was confirmed at 48 h. Consistent with the mRNA results, no significant effect of hypoxia was observed on NHE1 protein levels at 24 h (Fig. 3D).

### Effect of in vitro hypoxia on PASMC migration

**Figure 4:**
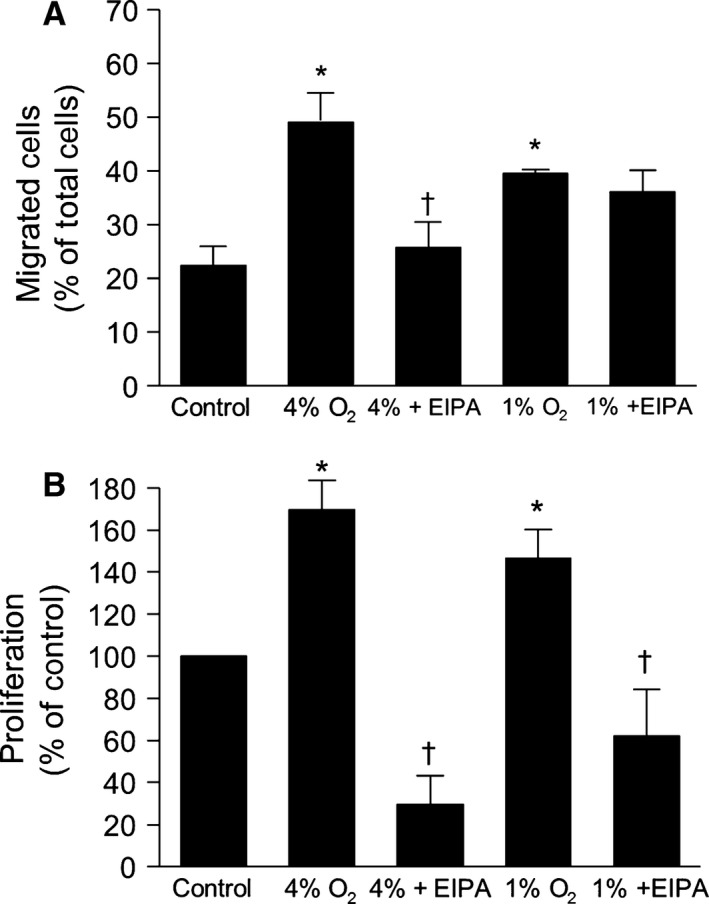
Inhibiting NHE attenuates PASMC migration and proliferation in response to in vitro hypoxia. (A) Bar graphs illustrating percent of migrated cells with and without EIPA (10^−5^ mol/L) incubated under control conditions (*n* = 15) or in either 4% O_2_ (*n* = 11 for untreated and *n* = 4 for EIPA) or 1% O_2_ (*n* = 4 for untreated and *n* = 3 for EIPA) for 24 h. (B) Bar graphs showing in vitro proliferation of PASMCs under control conditions (*n* = 23) and with and without EIPA (10^−5^ mol/L) incubated in either 4% O_2_ (*n* = 7 for untreated and *n* = 5 for EIPA) or 1% O_2_ for 96 h (*n* = 8 for untreated and *n* = 8 for EIPA). All bar graphs show mean ± SEM data.*Indicates significant difference from N value (*P *< 0.05); †indicates significant difference from untreated value at the same level of O_2_.

Exposing rat PASMCs to either 4% or 1% O_2_ increased migration rates (Fig. 4A), with PASMCs exposed to 4% O_2_ exhibiting greater migration than PASMCs exposed to 1% O_2_. EIPA significantly reduced the migration induced by 4% O_2_, but had minimal effect on migration in response to 1% O_2_. However, we noted that there was substantial loss of cells in PASMC exposed to 1% O_2_, with a decrease in total adhered cells to 202.3 ± 33.9 (*P *< 0.05 compared to control), which could lead to an overestimation of the resulting migration “rate.” In contrast, adherence in cells exposed to 4% O_2_ was 478.6 ± 55.9, similar to the value observed in nonhypoxic PASMCs.

### Effect of in vitro hypoxia on PASMC proliferation

In vitro exposure to either 4% O_2_ or 1% O_2_ significantly increased PASMC proliferation compared to control cells (Fig. 4B). Treatment with EIPA at the beginning of the exposure reduced proliferation to below basal values at both levels of hypoxia (4% and 1%).

### Role of ROCK in hypoxia‐induced PASMC proliferation and migration

**Figure 5:**
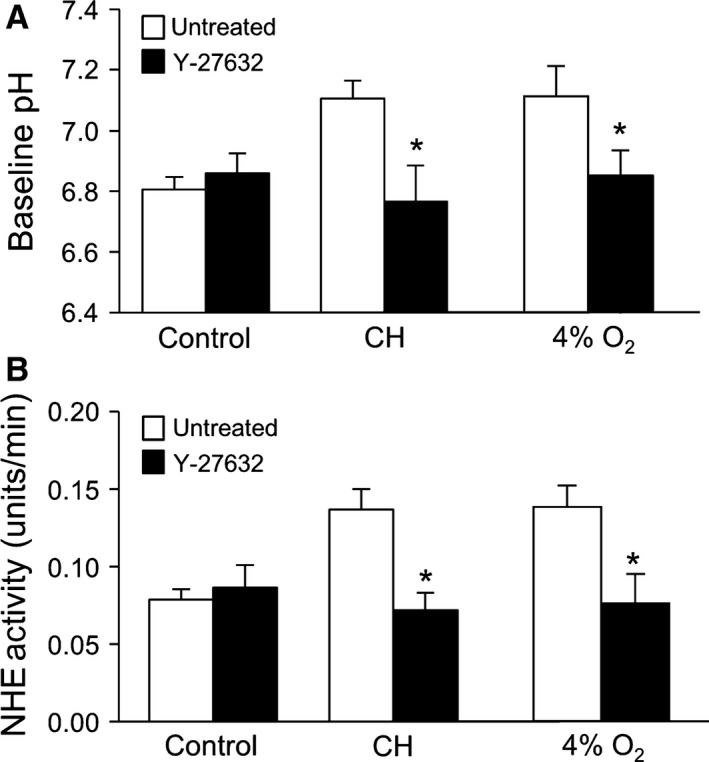
Inhibiting Rho kinase (ROCK) attenuates hypoxia‐induced changes in pH_i_ and NHE activity. (A) Bar graph showing baseline pH_i_ in PASMCs in the presence and absence of Y‐27632 (10^−5^ mol/L) under control conditions (*n* = 16 for untreated and *n* = 7 for Y‐27632), after isolation from chronically hypoxic (CH) animals (*n* = 8 for untreated and *n* = 5 for Y‐27632) or after exposure to 4% O_2_ (*n* = 6 for untreated and *n* = 4 for Y‐27632). (B) Bar graph showing NHE activity measured in the presence and absence of Y‐27632 in PASMCs under control conditions (*n* = 16 for untreated and *n* = 7 for Y‐27632), after isolation from CH animals (*n* = 8 for untreated and *n* = 5 for Y‐27632) or after exposure to 4% O_2_ (*n* = 6 for untreated and *n* = 4 for Y‐27632). All bar graphs show mean ± SEM data. *Indicates significant difference from untreated value within a group (*P *< 0.05).

**Figure 6:**
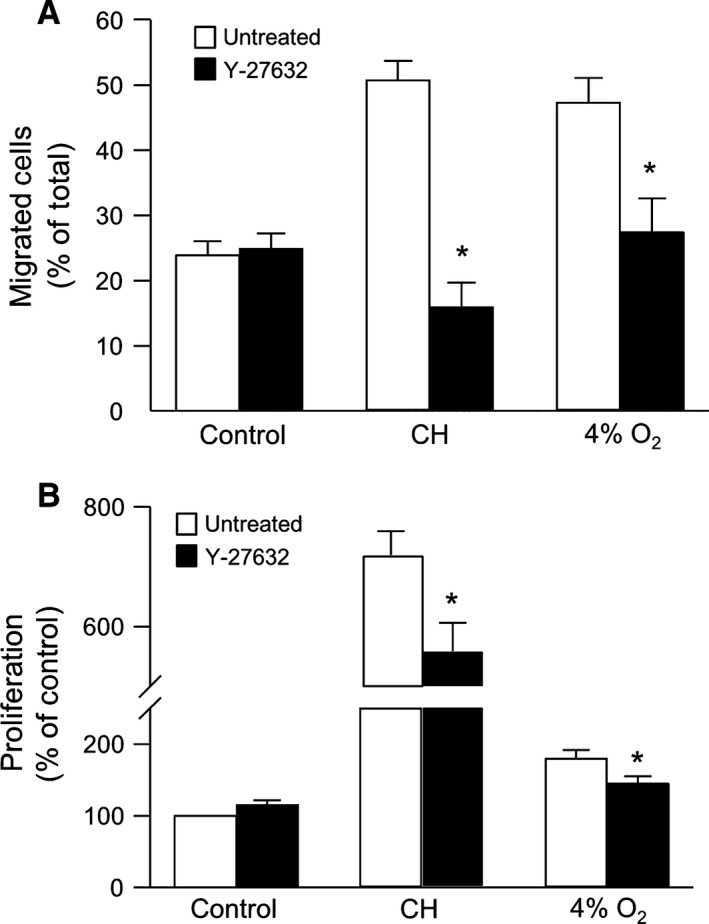
Inhibiting Rho kinase (ROCK) reduces hypoxia‐induced migration and proliferation. (A) Bar graph showing percent migrated PASMCs in the presence and absence of Y‐27632 (10^−5^ mol/L) under control conditions (*n* = 15 for untreated and *n* = 6 for Y‐27632), after isolation from chronically hypoxic (CH) animals (*n* = 5 for untreated and *n* = 4 for Y‐27632) or after exposure to 4% O_2_ (*n* = 11 for untreated and *n* = 7 for Y‐27632). (B) Bar graph showing proliferation of PASMCs measured in the presence and absence of Y‐27632 in PASMCs under control conditions (*n* = 23 for untreated and *n* = 8 for Y‐27632), after isolation from CH animals (*n* = 8 for untreated and *n* = 5 for Y‐27632) or after exposure to 4% O_2_ (*n* = 7 for untreated and *n* = 6 for Y‐27632). All bar graphs show mean ± SEM data. *Indicates significant difference from untreated value within a group (*P *< 0.05).

Since ROCK activation in PASMCs has been reported in response to hypoxia, we determined whether ROCK might contribute to the hypoxia‐induced enhancement of PASMC NHE activity, migration, and proliferation. In PASMCs treated with Y‐27632 (10 *μ*mol/L) for 30 min, basal pH_i_ and NHE activity were reduced in cells from chronically hypoxic, but not normoxic, rats (Fig. 5A and B). Similarly, Y‐27632 prevented the increase in both basal pH_i_ and NHE activity that occurred with in vitro exposure to hypoxia (4% O_2_; 24 h) (Fig. 5A and B). In the presence of Y‐27632, no significant effect on cell adherence was noted (473.3 ± 83.8) and Y‐27632 had little effect on migration (Fig. 6A) or proliferation (Fig. 6B) in cells from normoxic animals. However, treatment with Y‐27632 markedly reduced both migration and proliferation in PASMCs from both CH rats and cells exposed to 4% O_2_ in vitro (Fig. 6A and B).

### Effect of NHE1 deficiency on the development of pulmonary hypertension, pH homeostasis, and vascular remodeling

To investigate the role of NHE1 in the development of pulmonary hypertension, *Nhe1* ^*+/+*^ and *Nhe1* ^*−/−*^ mice were exposed to CH. Because loss of NHE1 results in neurological disturbances, including fatal seizures, approximately half of the *Nhe1* ^*−/−*^ mice in each exposure group died during the course of the experiment (*n* = 5 for normoxic and *n* = 6 for CH). The hemodynamic and physiologic measurements for these mice are summarized in Table 1. In surviving mice, mean RV pressure was similar in normoxic mice of both genotypes, whereas exposure to CH caused a significant increase in RVSP and mean RV pressure in *Nhe1* ^*+/+*^ mice only, with mean RV pressure in chronically hypoxic *Nhe1* ^*−/−*^ mice similar to those measured in normoxic mice. Chronically hypoxic *Nhe1* ^*+/+*^ mice had significantly higher RV weights than *Nhe1* ^*+/+*^ mice maintained under normoxic conditions (*P *< 0.05) and increased Fulton's index (*P *< 0.05). Loss of NHE1 was associated with smaller RV weights in normoxic mice although this difference did not reach statistical significance, and exposure to CH did not cause a statistically significant increase in RV weight in *Nhe1* ^*−/−*^ mice. LV+S weight tended to be lower in *Nhe1* ^*−/−*^ mice, but there was no statistically significant difference between any of the groups. The RV/LV+S ratio increased significantly in chronically hypoxic *Nhe1* ^*+/+*^ mice, but not in *Nhe1* ^*−/−*^ mice. Consistent with previous results (Yu et al. 2008), *Nhe1* ^*+/+*^ mice exposed to CH exhibited a marked increase in HCT. Interestingly, deficiency for NHE1 reduced CH‐induced polycythemia.

**Table 1:** Effect of chronic hypoxia on hemodynamic and physiologic parameters in *Nhe1* ^*+/+*^ and *Nhe1* ^*−/−*^ mice

Parameter	Normoxic		Hypoxic	
	*Nhe1* ^*+/+*^ (*n* = 6)	*Nhe1* ^*−/−*^ (*n* = 6)	*Nhe1* ^*+/+*^ (*n* = 6)	*Nhe1* ^*−/−*^ (*n* = 5)
Mean RV pressure (mmHg)	8.97 ± 2.6	9.6 ± 2.6	27.7 ± 2.1^a^	9.68 ± 1.3^b^
RV weight (mg)	21.7 ± 1.5	14.7 ± 1.6^a^	35.1 ± 2.5^a^	20.2 ± 4.8^b^
LV weight (mg)	91.3 ± 6.6	63.5 ± 7.3	105 ± 9.9	73.7 ± 6.4
RV/LV+S	0.24 ± 0.01	0.23 ± 0.01	0.34 ± 0.02^a^	0.27 ± 0.01^b^
HCT (%)	33.5 ± 2.2	36.5 ± 1.4	46.5 ± 0.6^a^	39.4 ± 2.5^b^

RV, right ventricle; LV, left ventricle; S, septum; HCT, hematocrit.

a *Significance from normoxic *Nhe1* ^*+/+*^.

b Significant difference from hypoxic *Nhe1* ^*+/+*^.

**Figure 7:**
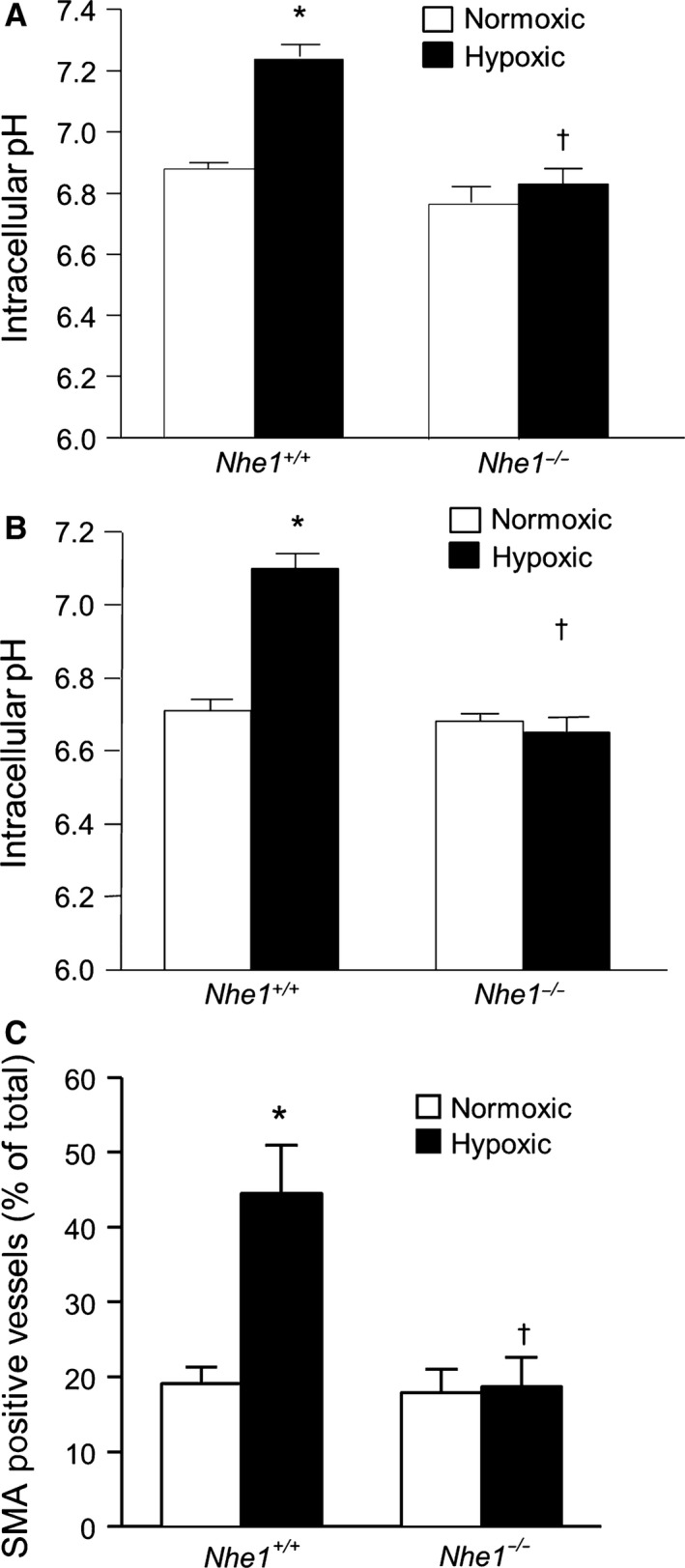
Deficiency for NHE1 prevents chronic hypoxia‐induced increases in pH homeostasis in mice. Bar graphs show baseline intracellular pH values measured in (A) KRBS solution (*n* = 3 for normoxic and *n* = 4 for hypoxic) and (B) HEPES‐buffered solution (*n* = 3 each) in PASMCs from normoxic and chronically hypoxic wild‐type (*Nhe1* ^*+/+*^) and NHE1‐deficient (*Nhe1* ^*−/−*^) mice. (C) Bar graphs showing percent actin‐positive vessels in *Nhe1* ^*+/+*^ and *Nhe1* ^*−/−*^ mice following exposure to normoxia or chronic hypoxia (*n* = 3 per group). All bar graphs show mean ± SEM data. *Indicates significant difference from normoxic *Nhe1* ^*+/+*^ value (*P *< 0.05); †indicates significant difference from hypoxic *Nhe1* ^*+/+*^ value.

The effect of NHE1 deficiency on PASMC pH homeostasis during normoxia and CH was determined by measuring pH_i_ during perfusion with bicarbonate‐containing extracellular solution (KRBS), as well as with bicarbonate‐free (HEPES‐buffered) extracellular solution, which eliminates the contribution from C1^−^/HCO_3_^−^ exchangers to pH changes. Consistent with our previous observations, exposure to CH caused a significant increase in PASMC pH_i_ in both KRBS (Fig. 7A) and HEPES‐buffered (Fig. 7B) extracellular solution. In PASMCs from normoxic *Nhe1* ^*−/−*^ mice, pH_i_ was similar to that measured in normoxic *Nhe1* ^*+/+*^ PASMCs, and exposure to CH did not increase pH_i_.

In lung sections stained with monoclonal antibody against smooth muscle cell specific *α*‐actin (SMA), the percentage of SMA‐positive small diameter (<100 *μ*m) vessels was determined (Fig. 7C). There was no difference in percent SMA‐positive vessels between genotypes exposed to normoxic conditions. *Nhe1* ^*+/+*^ mice exhibited an increase in the percentage of SMA‐positive vessels after CH exposure, while this increase was not seen in CH mice deficient for NHE1, suggesting a role for NHE1 during in vivo muscularization of small vessels upon CH exposure.

## Discussion

In this study, we found that ROCK‐mediated activation of NHE contributes to the hypoxic induction of PASMC migration and proliferation. PASMCs isolated from chronically hypoxic animals, or isolated from normoxic animals and exposed to hypoxia ex vivo, exhibited increased pH_i_, NHE activity, migration, and proliferation. In both cases, these effects could be blocked with NHE inhibitors. Inhibition of ROCK also normalized NHE activity, migration, and proliferation in response to both in vivo and in vitro hypoxic exposure. Moreover, right ventricular pressure, RV hypertrophy, vascular remodeling, and pH_i_ homeostasis were all normalized in chronically hypoxic mice that were deficient for NHE1. These data provide compelling evidence that an increase in NHE activity is an underlying mechanism controlling the growth and migration of PASMCs during hypoxia.

To our surprise, NHE1 deficiency also reduced hypoxia‐induced polcythemia. NHE1 is present in red blood cells (Sarangarajan et al. 1998), and recent reports indicate that loss of NHE1 is associated with hemolytic anemia (Wooden et al. 2011), suggesting a plausible basis for lower hematocrit in the *Nhe1* ^*−/−*^ mice. Regardless of the mechanism involved, the effect on red blood cell levels that we observed is at odds with the previous report by Yu et al. (Yu et al. 2008), where *Nhe1* ^*+/+*^ and *Nhe1* ^*−/−*^ mice exposed to CH had similar elevated hematocrits. The reason for the differences between our study and previously reported data is unclear, although the mice were on different genetic backgrounds. Further experiments are clearly needed to define the mechanism by which polycythemia was attenuated in *Nhe1* ^*−/−*^ mice.

A limitation of our study is that while the *Nhe1* ^*−/−*^ mice were backcrossed onto the C57B/6 background (~99.98% B6‐like) (Cox et al. 1997), they are slightly different from the C57B/6 WT mice used as controls. This concern is mitigated to some extent by the fact that development of pulmonary hypertension in response to hypoxia was also attenuated in *Nhe1* ^*−/−*^ mice on a different genetic (129SvJ/Black Swiss) background (Yu et al. 2008). Another limitation to the in vivo study is the severe seizure phenotype and short life span of the *Nhe1* ^*−/−*^ mice. A question that arises, which applies to both our study and the previously published report, is whether the seizure phenotype could have potentially contributed to the lack of pulmonary hypertension observed in the *Nhe1* ^*−/−*^ mice. While we cannot entirely rule out this possibility, in the study by Yu et al. (2008), mice with partial deficiency for (*Nhe1* ^*+/−*^ *)* exhibited an intermediate phenotype with respect to hypoxia‐induced pulmonary hypertension. The *Nhe1* ^*+/−*^ mice on the 129SvJ/Black Swiss background have been reported elsewhere to be fairly normal, living to adulthood, and capable of reproducing (Bell et al. 1999). Taken together, these findings would suggest that the seizure phenotype was unlikely to contribute to the lack of pulmonary hypertension in response to CH.

Previously, we demonstrated that exposure to CH increased basal pH_i_ and NHE activity in PASMCs from mice (Rios et al. 2005; Shimoda et al. 2006), and that these changes in PASMC pH regulation correlated with increased expression of NHE1. Several studies have demonstrated that NHE is important in regulating pH_i_ and cell proliferation in response to growth factors (Quinn et al. 1991, 1996; Madden et al. 2001), and our previous data demonstrated that NHE1 is the predominate isoform in PASMCs (Rios et al. 2005; Shimoda et al. 2006). Inhibitors of NHE were found to attenuate the development of hypoxic pulmonary hypertension; however, it was not clear whether this was a direct effect of inhibiting NHE activity in PASMCs, or was due to nonspecific effects of the drugs (Quinn et al. 1998). More recently, data from *Nhe1* ^*−/−*^ mice indicated that NHE1 plays a role in the development of hypoxic pulmonary hypertension, with the mice exhibiting reduced RV hypertrophy and vascular remodeling (Yu et al. 2008). However, in these studies, pH homeostasis was not examined. The data from our current study confirm that NHE1 is necessary for the development of pulmonary hypertension in response to CH. Furthermore, we demonstrate that loss of NHE1 also prevented the increase in PASMC pH_i_ and NHE activity observed in response to CH. As a whole, these results indicate that NHE1 plays a primary role in regulating PASMC pH_i_ homeostasis during CH.

The reduction in vascular remodeling observed in the chronically hypoxic mice lacking NHE1, coupled with reduced proliferation of PASMCs in response to growth factors when NHE is inhibited by amiloride analogs (Quinn et al. 1998) or siRNA approaches targeting NHE1 (Yu and Hales 2011), suggests that hypoxia might induce vascular remodeling via changes in pH_i_ homeostasis. To test this possibility, we used several approaches. First, we showed that PASMCs from chronically hypoxic rats exhibited similar changes in pH_i_, NHE activity, and NHE1 expression as were seen in PASMCs from chronically hypoxic mice. Next, we tested whether the cells from these animals exhibited differences in proliferation and/or migration. We found that both migration and proliferation were increased in PASMCs from chronically hypoxic rats, even when tests were performed under nonhypoxic conditions. This is consistent with our previous data demonstrating that the changes in pH_i_, NHE activity, and NHE1 expression persisted for at least 5 days after the cells were returned to normoxic conditions (Rios et al. 2005). The CH‐induced enhancement of PASMC migration and proliferation was absent when the cells were treated with an NHE inhibitor, which decreased pH_i_ in these PASMCs, further supporting a role for increased pH_i_ and NHE activity in mediating the effects of CH. Importantly, previous results examining hypoxia‐induced growth responses in PASMCs using siRNA directed against NHE1 were obtained in human PASMCs from conduit vessels exposed to severe hypoxic conditions (Yu and Hales 2011). However, there are reports demonstrating that the level of hypoxia to which PASMCs are exposed may induce different signaling pathways (Pisarcik et al. 2013), and that there can be distinct growth responses to stimuli based on location in the vascular tree. For example, BMP4 exerts growth stimulating effects in PASMCs from the distal lung, while in PASMCs from proximal vessels, BMP is growth suppressive (Yang et al. 2005). Similarly, differential effects of prostacyclin have also been observed (Wharton et al. 2000). Our results in this study show that moderate hypoxia (i.e., 4% O_2_) appears to induce similar proliferative responses in distal PASMCs as was previously observed in PASMCs from proximal vessels exposed to more severe hypoxia (Chen et al. 2009; Yu and Hales 2011; Green et al. 2012), and that NHE appears to mediate the response in both cases.

In vivo circulating factors could contribute to the development of a PASMC phenotype that expresses enhanced proliferation and migration capabilities. Thus, we tested whether exposure to ex vivo hypoxia could induce similar effects. We previously found that 48 h exposure to moderate hypoxia (4% O_2_) increased NHE1 expression in PASMCs (Shimoda et al. 2006; Pisarcik et al. 2013). In this study, we show that increases in pH_i_ and NHE activity occur within 24 h of hypoxia exposure to either moderate (4% O_2_) or severe (1% O_2_) hypoxia. Interestingly, NHE1 protein did not appear to be significantly increased with 24 h of moderate hypoxia; however, since total protein levels were measured, it is possible that the changes in NHE activity and pH_i_ seen at this earlier time point were due to increases in protein that were below the level of detection with immunoblot, due to increased surface expression of NHE1 or due to a change in the regulation of existing NHE1 proteins (i.e., differences in phosphorylation). With respect to the latter possibility, we recently demonstrated that acute application of ET‐1 increased NHE activity in PASMCs through ROCK (Undem et al. 2012). Since hypoxia has been shown to induce the production and/or secretion of ET‐1 and other vasoactive agents that activate ROCK (Tamm et al. 1998; Ambalavanan et al. 1999; Whitman et al. 2008) and ROCK is activated by CH (McMurtry et al. 2003; Girgis et al. 2007; Jernigan et al. 2008; de Frutos et al. 2011), we tested whether increased ROCK activation could be responsible for augmented activity of NHE. This appears to be the case, as ROCK inhibition reduced NHE activity in PASMCs from chronically hypoxic rats and normal PASMCs exposed to hypoxia ex vivo. NHE1 contains a number of phosphorylation sites, and has been shown to be regulated by several kinases (Li et al. 2013; Hendus‐Altenburger et al. 2014). Moreover, there are abundant data showing that NHE activity can be regulated by ROCK in a variety of cell types (Tominaga and Barber 1998; Tominaga et al. 1998; Cardone et al. 2005; Undem et al. 2012) and recently, Wallert et al. (2015) identified a ROCK1/2 phosphorylation site on NHE1 at serine 653 and showed that NHE1 is a direct phosphorylation target of ROCK. They further demonstrated that mutating this site rendered NHE1 incapable of being activated by lysophosphatidic acid (LPA) and prevented LPA‐induced migration in a fibroblast line. Thus, our results with hypoxia are consistent with this previous work. However, our findings differ from those in human proximal PASMCs, where NHE1 was found to be upstream of ROCK (Yu and Hales 2011). While these different findings could represent a potential feed‐forward system, with ROCK acting both upstream and downstream of NHE1, it is also possible that species or location of vessel from which PASMCs were derived also contribute.

It should be noted that in addition to being increased by exposure to hypoxia, some studies have reported basal activation of ROCK in pulmonary vascular smooth muscle (Jernigan et al. 2004; Weigand et al. 2011). Given these reports, it might be surprising that we found no effect of Y‐27632 in control cells with respect to any of the parameters tested. There are several possible explanations for these findings. We suspect that the lack of effect of ROCK inhibitor at baseline may be due to the fact that our cells were serum starved (0.5% FCS) for at least 24 h before they were plated for measurements, which were also conducted during serum starvation. Under these conditions, ROCK activation at baseline may be decreased to such an extent that additional inhibition of ROCK is unable to alter cell function. Along these lines, it is also possible that the level of basal ROCK activation in the pulmonary vasculature is below that required to active NHE and migration. Finally, another likely possibility is that ROCK activation is necessary, but alone not sufficient, to induce NHE activity, and that other factors induced by hypoxia are required to act in concert with ROCK activation to stimulate the exchanger.

While ROCK inhibition reduced both migration and proliferation in response to hypoxia, the effect was much more pronounced for migration. One question that might arise is whether a decrease in migration might have contributed to the reduction in proliferation. We believe that this is an unlikely scenario, as the assays for in vitro measurements were conducted in a manner to separate the effects of migration and proliferation as much as possible. For example, migration was measured as percent of cells on the bottom of the filter versus the total number of cells on both sides, which should have accounted for any proliferation that might occur. Similarly, since the proliferation assay was conducted using BrdU incorporation, we believe it is unlikely that a change in migration would alter these results significantly. Nonetheless, we recognize that cell movement, per se, is a critical process for both migration and proliferation, perhaps more so for migration. Thus, if the main effect of ROCK inhibition is to reduce cell movement, a more profound effect of ROCK inhibition on migration might not be unexpected.

Following 24 h of moderate hypoxic exposure, PASMCs exhibited a significant increase in migration, which was prevented by treatment with a NHE inhibitor. The slight increase in cell migration observed with exposure to severe hypoxia was not significantly different from the values observed in nonhypoxic cells nor was it different from cells exposed to hypoxia and NHE inhibitor. Inspection of the total cell counts on the transwell membranes before the removal of the nonmigrated cells indicated a sharp decrease in the number of cells present, suggesting that severe hypoxia may have negatively impacted either cell attachment or viability. Interestingly, we found that short‐term hypoxia (24 h) had either no effect on (4% O_2_) or caused a slight decreased (1%) in cell number when measured by cell counts (data not shown), indicating that the proliferative response to hypoxia in our cells required greater than 24 h to become evident. With respect to severe hypoxia, we speculate that this could be due, in part, to cell detachment or cell death in a certain population of the cells. These findings contrast with results reported in human PASMCs from proximal vessels, as severe hypoxia (1–2% O_2_) was found to induce proliferation of these cells within 24 h (Yu and Hales 2011). Whether these differences reflect variations between species or vessel location remains to be resolved. Nonetheless, at longer time points marked PASMC proliferation was noted in response to both 1 and 4% O_2_. Moreover, under both conditions, inhibition of NHE activity by inhibiting either NHE directly or via ROCK activity prevented the hypoxia‐induced increases in PASMC proliferation and migration.

In summary, the results from the current study demonstrate a clear role for ROCK‐induced activation of NHE in mediating alterations in pH_i_ homeostasis in PASMCs exposed to hypoxia. The fact that these changes could be induced by exposure to hypoxia ex vivo indicates that paracrine or circulating factors are not required for the response, although in vivo extrinsic factors may certainly act to modulate the response. Our results also indicate that the hypoxia‐induced changes in NHE activity and pH_i_ result in a promigratory and proliferative PASMC phenotype, suggesting an important contribution of NHE activation in the remodeling that occurs in hypoxia‐induced pulmonary hypertension. Our data also add to accumulating evidence supporting a central role for ROCK in promoting the pathogenesis and progression of pulmonary hypertension through control of a variety of pathways involved in both remodeling and vasoconstriction, and provide additional rationale for exploring ROCK and/or NHE1 inhibitors, especially those targeted specifically to the lung, as a future therapeutic option.

## Conflict of Interest

None declared.
